# A pandemic preparedness plan for common neurodegenerative diseases: a JPND advice report

**DOI:** 10.1002/alz.090661

**Published:** 2025-01-09

**Authors:** Marije J. Splinter, Emily J. Henderson, Yoav Ben‐Shlomo, Frank J. Wolters, Sirwan K.L. Darweesh, Pawel Sowa, Premysl Velek, Evelien I.T. de Schepper, M. Kamran Ikram, Silvan Licher

**Affiliations:** ^1^ Erasmus University Medical Center, Rotterdam, Zuid‐Holland Netherlands; ^2^ University of Bristol, Bristol, South‐West England United Kingdom; ^3^ Department of Neurology, Radboud University Medical Center, Nijmegen, Gelderland Netherlands; ^4^ Medical University of Bialystok, Bialystok, Podlaskie Poland; ^5^ Erasmus MC, Rotterdam, Zuid‐Holland Netherlands; ^6^ Erasmus MC University Medical Center, Rotterdam, Zuid Holland Netherlands

## Abstract

**Background:**

The expected seasonal recurrence of (new variants of) COVID‐19 and the emergence of other airborne infectious diseases underscore the need for a sustainable healthcare preparedness strategy. It is particularly essential to ensure equitable access to healthcare for individuals with chronic diseases, including dementia and Parkinson’s Disease. Moreover, it is vital to provide clinicians and researchers in the neurodegenerative disease fields with resources and infrastructure to ensure continuity of their work, even during a pandemic.

**Methods:**

We established an international collaboration and invited not only researchers and clinicians, but also patient representatives from the Netherlands, Poland, and the United Kingdom. We co‐created a pandemic preparedness plan building upon insights and experiences from a total of 25.000 participants from four population‐based studies during the COVID‐19 pandemic. Between March and November 2023, we organized two hybrid meetings in Bristol (United Kingdom) and Rotterdam (the Netherlands), and two online meetings. Key aspects of the plan included lessons learned in healthcare provision, healthcare utilization, and conducting research that could be applied during future pandemics.

**Results:**

During the group meetings, we outlined three fundamental elements of remote data collection during health crises: validated questionnaires, variated response options, and questionnaire testing within and beyond the research group. Additionally, we addressed the potential burden of participating in these studies for frail individuals, providing specific considerations about questionnaire length, readability, and the added value of supporting participants in completing a questionnaire. Finally, we made recommendations about data sharing, underlining the importance of legislative measures extending beyond the COVID‐19 pandemic, which are crucial for enabling successful integration and long‐term utilization of multiple data sources. Beside research‐related recommendations, we also shared clinical perspectives, including the risks of social isolation for individuals with dementia and Parkinson’s Disease, the dynamics of remote versus in‐person consultations, and the concept of moral injury among healthcare professionals who provided care for infected individuals during the pandemic.

**Conclusion:**

In this pandemic preparedness plan, we provide research and clinical recommendations and insights specifically tailored to the neurodegenerative disease fields. This establishes an essential framework for setting up new studies and safeguarding research and clinical practices when a new pandemic emerges.

